# PHF14 Promotes Cell Proliferation and Migration through the AKT and ERK1/2 Pathways in Gastric Cancer Cells

**DOI:** 10.1155/2020/6507510

**Published:** 2020-06-10

**Authors:** Yuzu Zhao, Jiang He, Yongsen Li, Man Xu, Xingzhi Peng, Jingxin Mao, Bo Xu, Hongjuan Cui

**Affiliations:** ^1^State Key Laboratory of Silkworm Genome Biology, Southwest University, Chongqing, China; ^2^Cancer Center, Medical Research Institute, Southwest University, Chongqing, China; ^3^Hospital of Southwest University, Southwest University, Chongqing, China

## Abstract

PHF14 is a new member belonging to PHD finger proteins. PHF14 is involved in multiple biologic processes including Dandy–Walker syndrome, mesenchyme growth, lung fibrosis, renal fibrosis, persistent pulmonary hypertension, and tumor development. This study aims to explore whether PHF14 plays an important role in gastric cancer. Here, PHF14 is indicated as a tumor promoter. The expression of PHF14 enhances no matter in clinical samples or in gastric cancer cells. High expression of PHF14 impairs survival of patients. Attenuation of PHF14 inhibits cell proliferation in gastric cancer cells. PHF14 downregulation inhibits the expression of cell cycle-related proteins, CDK6 and cyclin D1. Furthermore, silencing of PHF14 reduces the level of phosphorylated AKT as well as phosphorylated ERK1/2. Finally, downregulation of PHF14 in gastric cancer cells inhibits colony formation *in vitro* and tumorigenesis *in vivo.* These results indicate that PHF14 promotes tumor development in gastric cancer, so PHF14 thereby acts as a potential target for gastric cancer therapy.

## 1. Introduction

Gastric cancer is the fifth malignancy worldwide, and its mortality ranked the third worldwide [1]. According to the latest data from the global cancer observatory, there are 1,033,701 (5.7%) stomach cases among 18,078,957 new cancer cases in 2018 and 782,685 (8.2%) stomach deaths among 9,555,027 cancer deaths. Prevention and individualized treatment based on specific risk assessment are the optimal modalities to reduce the mortality of gastric cancer patients [2]. In addition, surgery plays an indispensable role in gastric cancer therapy [3, 4]. However, neither prevention and individualized treatment nor surgery is favorable for patients with advanced and metastatic stomach cancer [5, 6]. Therefore, it is urgent to explore novel biomarkers to provide more therapeutic choices for patients with advanced gastric cancer.

PHD (plant homeodomain) finger proteins are conserved in eukaryotes from yeast to humans and play important roles in multiple biological events [7–9]. PHD proteins bind to the chromatin and then influence the expression of epigenetic genes by modulating the structure of the chromatin [10–12]. PHD fingers act as the structure basis to recognize various epigenetic genes, such as *H3K4me3, H3K4*, *H3K9me3*, and *H3K14ac* [13–18]. PHF14 is a member belonging to the PHD family, which consists of 4 PHD domains, and PHF14 interacts with the histone through its PHD1 and PHD3 domains [19]. As a newly identified protein, there are some functions of PHF14 in previous studies as given in the following. PHF14 is firstly identified as a colon tumor suppressor [20]. PHF14 is related to Dandy–Walker syndrome as well [21]. PHF14 acts as a transcription inhibitor of the platelet-derived growth factor receptor-*α* (PDGFR*α*) and then regulates mesenchyme growth, indicating that PHF14 is a potential target for lung fibrosis treatment [22]. PHF14 also plays an essential role in BTC. Downregulation of PHF14 accelerates growth of BTC cells [23]. PHF14 inhibits renal fibrosis after kidney injury induced by folic acid [24]. Depletion of PHF14 inhibits cancer cell proliferation and tumorigenesis in lung cancer [25]. PHF14 is involved in persistent pulmonary hypertension. Knocking down of PHF14 results in lethal respiratory failure in animal experiments [19]. These studies indicate that PHF14 plays important roles in multiple biological processes as well as tumor development; however, the role of PHF14 in gastric cancer remains unclear. This study is mainly focused on the importance of PHF14 in gastric cancer cells.

## 2. Materials and Methods

### 2.1. Reagents and Antibodies

Rabbit reinforced polymer detection system for immunohistochemistry (IHC, #PV-9001) was purchased from ZSGB-BIO (Beijing, China). 3-(4, 5-Dimethylthiazol-2-yl)-2, 5-diphenyltetrazolium bromide (MTT, #M5655), bromodeoxyuridine (BrdU, #B5002), and agarose with low gelling temperature (#SLBS5420) were purchased from Sigma-Aldrich (Shanghai, China). Antibody against PHF14 (#24787-1-AP) was purchased from Proteintech (Wuhan, China), and CDK6 (#3136) and Cyclin D1 (#2978) antibodies were purchased from Cell Signaling Technology (Shanghai, China). P-AKT (#A5030), P-ERK1/2 (#A5036), and Beta Tubulin (#A5032) antibodies were obtained from Bimake group (Shanghai, China).

### 2.2. Cell Lines and Culture

Human gastric cancer cell lines, SGC-7901, HGC-27, and MKN-45, human gastric mucosa cell line GES-1, and 293FT cell lines were obtained from American Type Culture Collection (ATCC, Manassas, VA, USA). HGC-27 and MKN-45 cells were cultured in RPMI-1640 (BI, Israel). This medium was supplemented with 10% fetal bovine serum (FBS, BI, Israel) and 1% penicillin and streptomycin (P/S, Gibco, New York, NY, USA). Gastric cancer cells mentioned above were cultured in 5% CO_2_ at 37°C.

### 2.3. Plasmid Transfection and Infection

Human PHF14 short hairpin RNAs (#1, TRCN0000019309; #2, TRCN0000019310) were purchased from Sigma-Aldrich and cloned into the PLKO.1 vector. Sequences used were presented as follows: shPHF14 #1, 5′-CCGGCGCATGATTCAAATTCAGGAACTCGAGTTCCTGAATTTGA ATCATGCGTTTTT-3′; shPHF14 #2, 5′-CCGGCCTGTAGTGATTCTGAAGAAACTCGAGTT TCTTCAGAATCACTACAGGTTTTT -3′. Packaging plasmids, including pLP1, pLP2, and VSVG, together with shRNA plasmid, were transfected into 293FT cells using Lipofectamine 2000 reagent (Invitrogen, #11668019) following the manufacturer's protocol. After 48 h, supernatants were collected and then infected with gastric cancer cells with polybrene.

### 2.4. IHC Assay

For IHC analysis, according to previous protocol [26], after deparaffinization, hydration, and antigen retrieval, the samples were then quenched for endogenous peroxidase and exposed to an antibody against PHF14 at 4°C overnight (1 : 100). According to the manufacturer's instruction, the samples were incubated with a signal enhancement solution for 20 min and then incubated with the horseradish peroxidase-linked secondary antibody for 20 min as well. Before capturing by a microscope, samples were counterstained using 3,3′-diaminobenzidine and hematoxylin for visualization.

### 2.5. Patients' Data Analysis

Patients' data were analyzed in the GEPIA database [27] for gene expression and the KM-plotter database [28] for prognosis, respectively. *p* values were obtained from the database as well.

### 2.6. Western Blot

Western blot analysis was performed as previously described. In detail, cells were harvested and then lysed using an RIPA lysis buffer (Beyotime, #P0013B) added with phenylmethanesulfonyl fluoride (PMSF, Beyotime, #ST506). After concentration measurement, and lysates were denatured. Proteins were separated by the 10% SDS-PAGE gel. After transferring proteins to the membrane, blocking membranes in 5% skim milk, membranes were incubated with indicated antibodies at 4°C overnight. Membranes were then exposed to the horseradish peroxidase-conjugated secondary antibody at room temperature for 2 h. Proteins were finally detected by an ECL system (Beyotime, #P0018AS) and captured by the ProXima chemiluminescence gel imaging system (Isogen, De Meern, Utrecht, Netherlands).

### 2.7. Cell Viability Assay

MTT assay was performed to detect the cell viability as previously described [29].

### 2.8. Proliferation Assay

2 × 10^4^ gastric cancer cells were seeded in 24-well plates and then incubated at 37°C in an incubator overnight. After incubating with 10 *μ*g/ml BrdU (Sigma-Aldrich, USA) at 37°C for 2 h, cells were fixed with 4% paraformaldehyde for 15 min. Cells were then treated with 2 M HCL and then with 0.3% TritonX-100 before blocked with 10% goat serum. Successively, cells were exposed to the BrdU antibody (Abcam, Cambridge, USA) and then with the secondary antibody (1 : 2000). Before captured by a microscope, cells were counterstained with Hoechst (1 : 2000).

### 2.9. Migration and Invasion Assay

Migration and invasion assays were performed as described previously. In brief, cells were seeded in 24-well Millicell Hanging chambers (Merck, Millipore) with 8.0 *μ*m pore size. For the invasion assay, the insert was covered by matrigel. Cells were cultured in a serum-free medium in the upper chamber, and in the lower chamber, the mediums were supplemented with 10% FBS. After culturing at 37°C for 24 h for the migration assay and 48 h for the invasion assay, respectively, cells were stained with crystal violet and captured.

### 2.10. Colony Formation Assay

A soft agar assay was performed to assess the colony formation ability of gastric cancer cells. 0.6% agarose diluted in 1.5 ml 2 × RPMI-1640 added with 20% FBS and 2% P/S was added into 6-well plates as a base layer. 1000 cells in 1 ml RPMI-1640 containing 0.3% agarose were added onto the base layer. After 15–20 days of culturing, colonies were captured by a microscope and then stained with MTT for scan.

### 2.11. Tumor Xenograft Assay

4-week-old female nude mice were purchased and raised in an SPF room in a standard condition. 1 × 10^6^ MKN-45 cells infected with shGFP and shPHF14 were subcutaneously injected to flanks of mice (cells infected with shGFP were on the left side, and cells infected with shPHF14 were injected on the right side of each mouse). Tumor volumes were measured by calipers and then calculated with the following formula: volume = (*π*/6) × length × width^2^ every three days. After 23 days, the mice were euthanized, and tumors were removed and weighted.

### 2.12. Statistical Analysis

Microsoft Excel was used for statistics analysis. Quantitative data were presented as the mean ± SEM and analyzed using unpaired two-tailed *t*-tests. Significant difference was computed by GraphPad and R software. *p* values <0.05 (^^*∗*^^) and <0.01 (^*∗∗*^) were considered statistically significant.

### 2.13. Ethics Statement

All animal studies were approved by the Animal Ethics Committee of Southwest University and performed humanly in accordance with the guidelines of the Care and Use of Laboratory Animals (Ministry of Science and Technology of China, 2006). Informed consent was obtained for all patients who supplied the gastric cancer samples. The use of the human tissues were approved by an ethics committee.

## 3. Results

### 3.1. PHF14 Is Highly Expressed in Gastric Cancer

**Figure 1:**
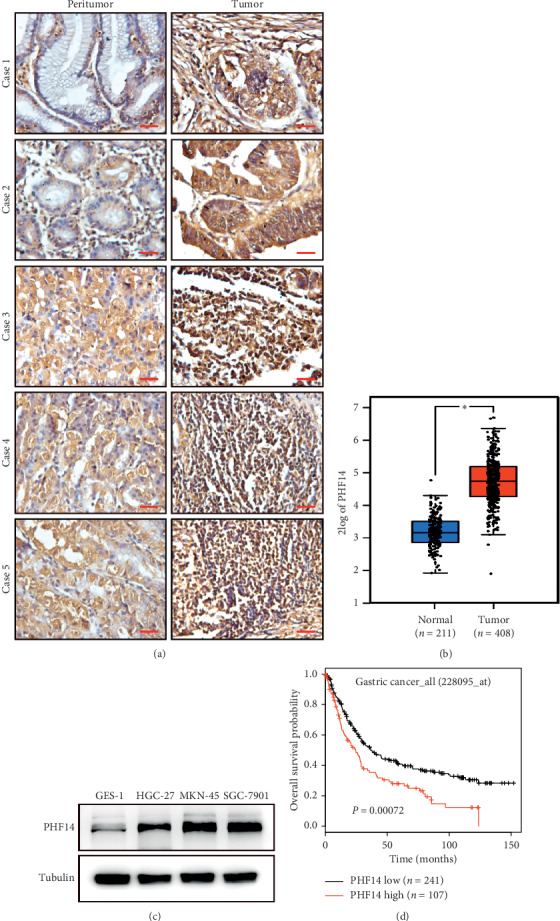
PHF14 is highly expressed in gastric cancer. (a) IHC of PHF14 in gastric cancer samples as well as in peritumor samples. (b) The expression of PHF14 in normal samples and in neoplastic samples from the GEPIA database. (c) The expression profile of 3 gastric cancer cell lines and a gastric epithelial cell line. (d) Prognostic analysis of PHF14 in the KM-plotter database.

In order to determine the role of PHF14 in gastric cancer, an IHC assay was performed and results revealed that PHF14 is highly expressed in gastric cancer samples compared with adjacent peritumor gastric tissues (Figure 1(a)). We then analyzed the expression of PHF14 in gastric patients using the GEPIA database, and results showed that the expression of PHF14 enhanced in 408 neoplastic samples compared with 211 normal samples (Figure 1(b)). Further, we checked the expression of PHF14 in 3 gastric cancer cell lines as well as in a gastric epithelial cell line. PHF14 is consistently overexpressed in cancer cells in comparison with normal gastric mucosa cells (Figure 1(c)). The results of prognostic analysis indicated that patients with high expression of PHF14 present poor prognosis (Figure 1(d)).

### 3.2. PHF14 Is Involved in Cell Proliferation in Gastric Cancer Cells

**Figure 2:**
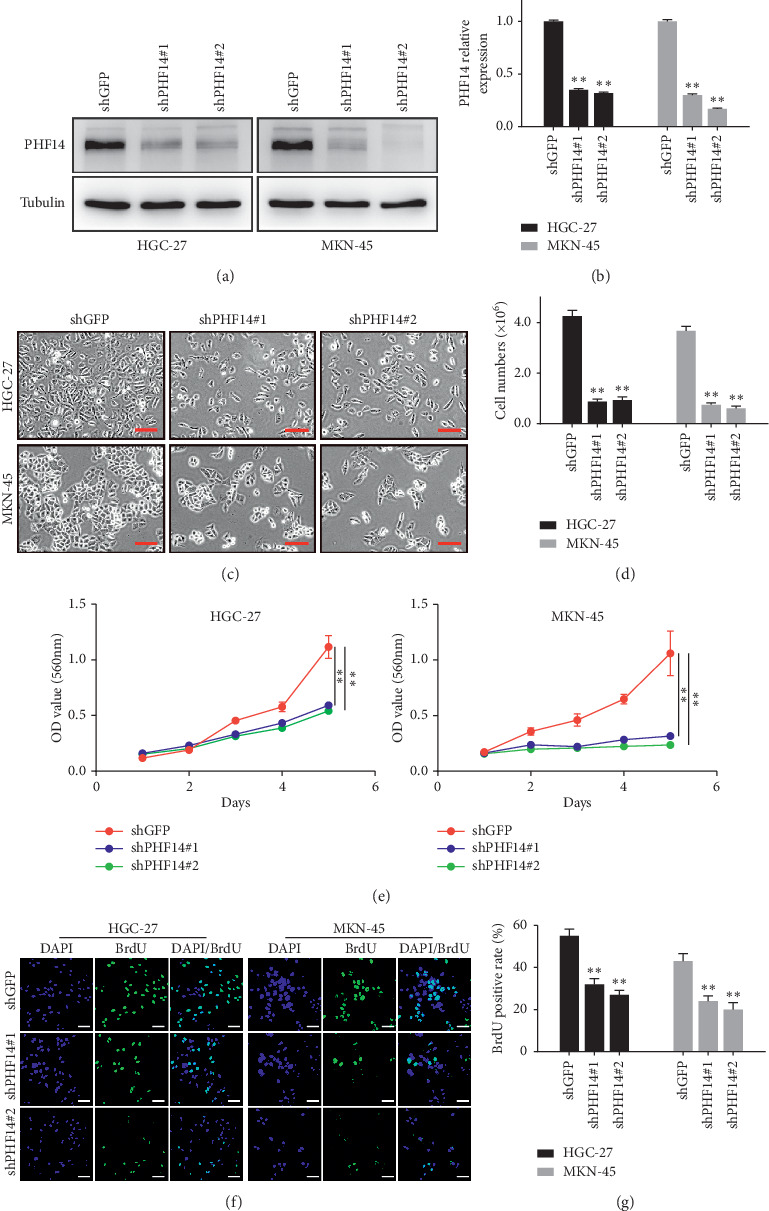
PHF14 promotes cell proliferation in gastric cancer cells. (a) The expression of PHF14 after knocking down of PHF14 in gastric cancer cells. (b) Densitometry of western blot in panel (a). (c) The morphology of gastric cancer cells after knocking down of PHF14. (d) Cell numbers of gastric cancer cells after downregulating PHF14. (e) Viability of gastric cancer cells knocking down PHF14. (f) BrdU-positive gastric cancer cells after knocking down of PHF14. (g) Quantification of BrdU-positive gastric cancer cells knocking down PHF14 in panel (f). All data are used as mean ± SD, and significant difference was tested by Student's *t*-test. ^*∗*^*p* < 0.05, ^*∗∗*^*p* < 0.01, ^*∗∗∗*^*p* < 0.001, and *p* value <0.05 were considered as statistically significant.

To investigate the effects of PHF14 in gastric cancer cells, two pairs of shRNAs against PHF14 were designed and shGFP was used as a control. PHF14 was knocked down in two gastric cancer cell lines, HGC-27 and MKN-45 (Figures.  2(a) and 2(b)). After downregulating of PHF14, gastric cancer cells showed significant morphological changes, and cells are decreased obviously in both HGC-27 and MKN-45 cells (Figures 2(c) and 2(d)). An MTT assay was then performed to evaluate the viability of HGC-27 and MKN-45 cells. Results indicated that the viability of cells knocking down PHF14 decreased compared with the shGFP group (Figures 2(e)). A BrdU assay was also performed to detect the DNA synthesis in gastric cancer cells. Consistently, DNA synthesis reduced in PHF14 downregulated cells in comparison with the shGFP group (Figures 2(f) and 2(g)).

### 3.3. PHF14 Is Required for Cell Migration in Gastric Cancer Cells

**Figure 3:**
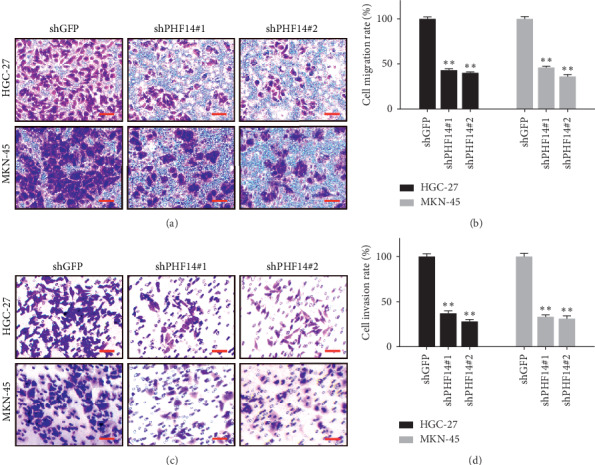
PHF14 promotes the migration and invasion in gastric cancer cells. (a) Migration of gastric cancer cells after knocking down of PHF14, (b) quantification of migration in panel (a), (c) invasion of gastric cancer cells after knocking down of PHF14, and (d) quantification of invasion in panel (c).

In order to investigate whether PHF14 is involved in migration and invasion in gastric cancer cells, a migration assay and invasion assay were performed. After knocking down of PHF14 in HGC-27 and MKN-45, cells migrated slower than cells from the shGFP group (Figures 3(a) and 3(b)). Matrix gel was then added to detect invasion. Cell downregulation of PHF14 invaded slower than cells from the shGFP group as well (Figures 3(c) and 3(d)).

### 3.4. PHF14 Is Required for Cell Cycle through AKT and ERK1/2 Pathways in Gastric Cancer Cells

**Figure 4:**
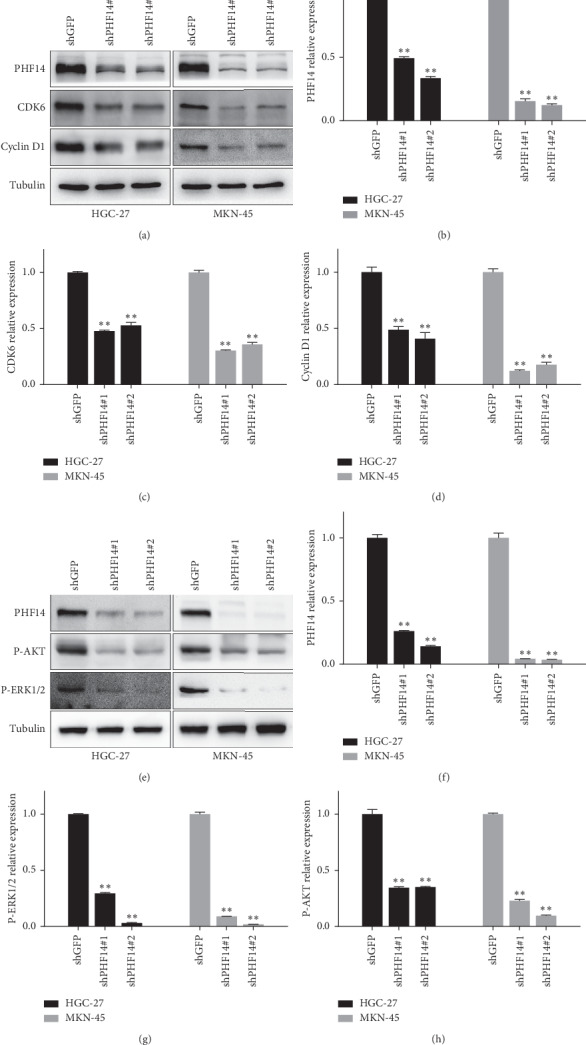
PHF14 is required for cell cycle through AKT and ERK1/2 pathways in gastric cancer. (a) The expression of CDK6 and cyclin D1 in gastric cancer cells after knocking down of PHF14, (b) densitometry of PHF14 in panel (a), (c) densitometry of CDK6 in panel (a), (d) densitometry of cyclin D1 in panel (a), (e) the expression of P-AKT and P-ERK1/2 in gastric cancer cells after knocking down of PHF14, (f) densitometry of PHF14 in panel (e), (g) densitometry of P-AKT in panel (e), and (h) densitometry of P-ERK1/2 in panel (e). All data are represented as mean ± SD, and significant difference was tested by student's *t*-test. ^*∗*^*p* < 0.05, ^*∗∗*^*p* < 0.01, ^*∗∗∗*^*p* < 0.001, and *p* value <0.05 were considered as statistically significant.

We further examined cell cycle-related proteins to assess whether knocking down of PHF14 inhibits cell proliferation through inducing cell cycle arrest. CDK6 and cyclin D1 are proteins which are essential for G1/S phase transition. As expected, CDK6 and cyclin D1 were decreased in both HGC-27 and MKN-45 gastric cancer cells after the knock down of PHF14 (Figures 4(a)–4(d)). PI3K/AKT and Ras-ERK are important signaling pathways involved in regulating cell proliferation [30]. Phosphorylated AKT and phosphorylated ERK1/2 were checked as well. Results showed that knocking down of PHF14 inhibits the activation of AKT and ERK1/2 (Figures 4(e)–4(h)). These results remind us that PHF14 may promote cell proliferation through the AKT and ERK1/2 pathways.

### 3.5. PHF14 Contributes to Colony Formation *In Vitro* and Tumor Formation *In Vivo*

**Figure 5:**
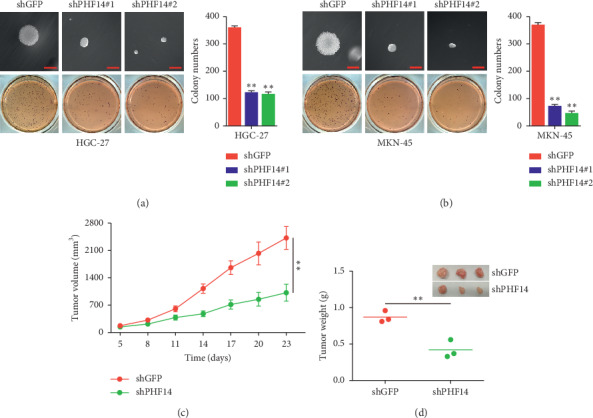
PHF14 promotes clonogenicity and tumorigenesis in gastric cancer cells. (a) Colony-formation ability of HGC-27 cells after knocking down of PHF14. (b) Colony-formation ability of MKN-45 cells after knocking down of PHF14. (c) Tumor volume of indicated mice. (d) Tumor weight and photograph of indicated mice. All data are used as mean ± SD, and significant difference was tested by Student's *t*-test. ^*∗*^*p* < 0.05, ^*∗∗*^*p* < 0.01, ^*∗∗∗*^*p* < 0.001, and *p* value <0.05 were considered as statistically significant.

To further confirm the role of PHF14 in gastric cancers, the soft agar assay and the subcutaneous xenograft experiment were performed, respectively. Cells knocking down PHF14 showed a decline in both colony number and colony volume (Figures 5(a) and 5(b)). The subcutaneous xenograft experiment showed that tumors formed by cells knocking down PHF14 grow slower than those in of the shGFP group not only in tumor volume, but also in tumor weight (Figures 5(c) and 5(d)).

## 4. Discussion

Gastric cancer is a malignancy initiating from the gastric epithelium, and the mortality of gastric cancer ranks the third in all cancer worldwide [31]. The best approaches to reduce the mortality in gastric cancer are prevention and early detection. Nevertheless, most patients suffering from gastric cancer are usually diagnosed at the advanced stage. Besides, patients treated with surgery always develop a relapse. Neoadjuvant treatment such as radiotherapy [32], chemoradiotherapy [33], and chemotherapy [34] is available in the advanced gastric cancer, but patients undergoing these treatments develop poor prognosis, and they do not survive more than 1 year. Targeted therapy with a specific biomarker might improve the prognosis of gastric cancer patients [2].

This study indicates that PHF14 serves as a tumor promoter in gastric cancer. PHF14 is highly expressed in tumor tissues and in cell lines, which reminder us that PHF14 may be regarded as a biomarker in diagnosis of gastric cancer. Moreover, higher level of PHF14 indicates lower survival in patients suffering from gastric cancer. This suggests that PHF14 can be utilized as a prognostic marker in gastric cancer. Furthermore, knocking down of PHF14 inhibits cell proliferation as well as tumorigenesis in gastric cancer. These findings intensify the tumor-accelerating function of PHF14 in gastric cancer. PHF14 is therefore regarded as a promising therapeutic target in gastric cancer. PI3K/AKT and Ras-ERK are important signaling pathways involved in regulating cell proliferation. This study further demonstrates that AKT and ERK1/2 pathways are involved in the inhibition of cell proliferation induced by PHF14 downregulation. Knocking down of PHF14 inhibits the activation of AKT and ERK1/2. Because of the essential role of AKT and ERK1/2 pathways in cell proliferation as well as in cancer development, inhibitors against PHF14 can be explored for gastric cancer therapy.

According to a previous study, the PHD finger family mainly consists of histone-binding proteins [19, 35], and some members of this family participate in transcriptional regulation [36]. PHD finger proteins are generally discovered as tumor promoters, and they are highly expressed in multiple tumors [25, 37, 38]. Consistent with these studies, our study indicates that PHF14 is a tumor promoter in gastric cancer, which enriches the role of PHD finger proteins in tumor acceleration. Some study illustrates that PHD finger proteins generally promote proliferation and migration in cancer cells [25, 37], which is also consistent with our results. A latest study indicates that silencing PHF14 induces apoptosis in glioblastoma cells [39]; however, there is no apoptosis in gastric cancer cells after knocking down of PHF14, which remind us that PHF14 plays different roles in different cancers. The different role of PHF14 may be induced by activation of different pathways. Studies about PHF14 in cancer development is still limited. It is necessary to explore the role of PHF14 in various processes such as epigenetic regulation, chemoradiotherapy resistance, and tumor metabolism. With the constant-depth study, PHF14 will present more clinical values.

## 5. Conclusion

This study shows that PHF14 accelerates tumorigenesis in gastric cancer. Meanwhile, this study indicates that PHF14 serves as a potential biomarker for tumor diagnosis, prognosis, and therapy in gastric cancer.

## Data Availability

The data used to support the findings of this study are included within the article.
